# Composition of the Gut Microbiota in Older Adults Residing in a Nursing Home and Its Association with Dementia

**DOI:** 10.3390/nu18030505

**Published:** 2026-02-02

**Authors:** Giada Sena, Francesco De Rango, Elisabetta De Rose, Annamaria Perrotta, Maurizio Berardelli, Angelo Scorza, Bonaventura Cretella, Giuseppe Passarino, Patrizia D’Aquila, Dina Bellizzi

**Affiliations:** 1 Department of Biology, Ecology and Earth Sciences, University of Calabria, 87036 Rende, Italy; giada.sena@unical.it (G.S.); francesco.derango@unical.it (F.D.R.); elisabetta.derose@unical.it (E.D.R.); annamaria.perrotta@unical.it (A.P.); giuseppe.passarino@unical.it (G.P.); patrizia.daquila@unical.it (P.D.); 2 Azienda Sanitaria Provinciale di Cosenza, Via Alimena 8, 87100 Cosenza, Italy; maurizio.berardelli@aspcs.it; 3 Villa Ermelinda, 88842 Cutro, Italydottcretella@inwind.it (B.C.)

**Keywords:** gut microbiota, aging, older adults, nursing home, dementia

## Abstract

**Background**: The human gut microbiota plays a pivotal role in maintaining health throughout the lifespan, and age-related alterations in its composition and diversity have been implicated in numerous chronic and neurodegenerative conditions. However, the combined effects of aging, dementia, and shared living environments on gut microbial communities remain incompletely understood. **Methods**: This study included 56 older adults residing in a nursing home, of whom 29 had been diagnosed with dementia. Gut microbiota composition was characterized by 16S ribosomal RNA (rRNA) gene sequencing. Microbial diversity was assessed using alpha- and beta-diversity metrics, and differences in amplicon sequence variants (ASVs)/features were determined. Analyses adopted some covariates as potential confounders variables including age, sex, frailty status, drug use, and time spent in the nursing home. **Results**: Alpha diversity was significantly higher in older adults compared with younger, while beta-diversity analyses revealed distinct microbial community structures between age groups. In older individuals, *Bacteroidota* and *Proteobacteria* were the most abundant phyla, whereas *Firmicutes* and *Actinobacteriota* declined with advancing age. Notably, older adults exhibited an increased relative abundance of *Euryarchaeota*, a phylum encompassing *Archaea*, predominantly methanogens involved in anaerobic carbon dioxide reduction to methane. In subjects with dementia, marked compositional shifts were detected, resulting in a distinct microbial signature. Dementia was associated with a significant enrichment of *Actinobacteriota*, *Euryarchaeota*, and *Proteobacteria*, alongside a depletion of *Bacteroidota* and *Firmicutes*. Overall, different bacterial genera mostly belonging to the *Firmicutes* phylum were associated both with aging and dementia. **Conclusions**: Results show age-related remodeling of the gut microbiota, with a stable core of common taxa and distinct individual-specific signatures. These shifts reflect both host factors and life-long environmental conditions. Dementia-related changes seem to correlate with increased inflammatory species, thus suggesting the effect of vulnerability in microbiota changes in subjects sharing living environment and diet.

## 1. Introduction

Aging is a genetically determined and environmentally modulated process that involves changes in the dynamics of biological, environmental, behavioral, and social processes [1]. Alongside different hallmarks of aging reported in the literature over the years, the rapid development of next-generation sequencing technologies has significantly contributed to demonstrating a strong association between a balanced gut microbiota and healthy aging [2,3,4,5]. On the one hand, aging exerts physiological effects on both the host and the microbiota, and on the other hand, host-microbiota interactions may also impact aging. Gut microbiota may modulate aging-related changes in innate immunity, sarcopenia, and cognitive function, all of which are elements of frailty [6,7,8,9,10,11]. There is no chronological threshold or age at which the composition of microbiota undergoes a sudden alteration; changes in the composition and function rather occur gradually with time and between individuals and are influenced by genetics and numerous lifestyle factors, including diet, physical activity, smoking, sleep quality, mental health, and medication [12,13]. Some studies investigated microbiota composition in residents of a nursing home by considering that a shared and well-defined living environment offers a unique opportunity to distinguish the role of the above factors on the gut microbiota [14,15,16]. Older people often exhibit decreased chewing ability, tooth loss, and diminished taste perception, which can affect appetite and dietary choices and ultimately impact their gut microbiota composition. What is more, they have reduced intestinal function, which may lead to constipation and affect digestion, nutrient absorption, and immune function [7,17,18,19]. Although variations have been observed across study populations, sample sizes, methodologies, and designs, the abundance of core commensals such as *Bifidobacterium* and *Firmicutes* is reduced in older adults. At the same time, an expansion of *Proteobacteria*, such as *Escherichia*, and *Bacteroidetes*, as well as opportunistic microbes such as *Fusobacterium*, *Parabacteroides*, and *Ruminococcaceae*, which are present at low abundance in healthy young individuals, is observed [20,21,22]. An imbalance in gut microbiota, known as dysbiosis, is often associated with impaired functionality and a shift in the microbiota’s metabolic profile, including changes in the bioavailability of metabolites, such as short-chain fatty acids (SCFAs) and secondary bile acids [23]. The *Firmicutes*/*Bacteroidetes* ratio, which undergoes an increase from birth to adulthood and is further altered with advanced age, is crucial to produce SCFAs [24,25,26].

Studies conducted over the past fifteen years highlight a direct link between the density and species diversity of the gut microbiota and several pathological disorders, including diabetes, obesity, and cardiovascular disease. Furthermore, it is well known that the gut microbiota can communicate with the brain and modulate behavior by regulating the activation state of the hypothalamic–pituitary–adrenal (HPA) axis and activating the vagus and adrenergic nerves, thus influencing the course of various age-related neurological disorders such as Alzheimer’s disease (AD), Parkinson’s disease (PD), depression, or multiple sclerosis (MS), as well as a whole series of non-Alzheimer’s and non-Parkinson’s dementias through various mechanisms that regulate peripheral neurotransmitters, metabolites, and immune signaling molecules [27,28,29]. Cattaneo et al. have demonstrated that Alzheimer’s disease patients with cognitive impairment and amyloidosis exhibit an increase in proinflammatory bacteria (*Escherichia*/*Shigella*) and a reduction in anti-inflammatory species (*Eubacterium rectale*), with microbial compositions correlated with the pathological biomarkers β-amyloid and tau [30,31]. It was also reported that women, presenting two or more subjective memory complaints (SMCs) associated with multiple pathological features of Alzheimer’s disease, exhibited alterations in gut microbiota composition. Notably, these changes included an increased abundance of the *Deltaproteobacteria*–*Desulfovibrionales*–*Desulfovibrionaceae* lineage within the phylum *Proteobacteria* [32]. In Parkinson’s disease, an imbalance characterized by an increase in potentially pathogenic bacteria and a reduction in butyrate producers (*Faecalibacterium*, *Coprococcus*, *Blautia*, and *Roseburia*) is associated with clinical severity and motor deterioration, likely through mechanisms of increased intestinal permeability and inflammation [33,34,35,36]. Other forms of dementia, including frontotemporal dementia, dementia with Lewy bodies, and vascular dementia, also show specific associations with microbial taxa (*Melainabacteria*, *Rhodospirillaceaem, Phascolarctobacterium*, and *Rhodospirillales*) that may act as risk or protective factors, suggesting a significant role for the gut microbiota in modulating neuroinflammation and neurodegenerative pathogenesis [37,38,39,40].

The primary objective of this study was to comprehensively characterize the diversity and taxonomic composition of the gut microbiota in older adults residing in a nursing home. Considering the high prevalence of dementia within this population and the increasing evidence supporting the role of the gut–brain axis, the study further aimed to investigate the association between gut microbiota profiles and dementia status.

## 2. Materials and Methods

### 2.1. Characteristics of the Participants to Study

The study was carried out on 56 older adults (mean age 83.6 ± 7.7) residing in the nursing home “Villa Ermelinda” located in Cutro (KR, Southern Italy) over an average period of about three years. Thirty-six young healthy subjects (mean age 42.36 ± 13.23) dwelling in the community were also analyzed as controls. The Territorial Ethics Committee of the Calabria Region (Authorization protocol number 79/2023) approved the study protocol that was performed in strict accordance with the Declaration of Helsinki. Following written informed consent, all subjects underwent a multidimensional geriatric assessment, reporting their state of health and morbidity, and taking medications. Exclusion criteria were the following: (i) therapy with antibiotics 3 months before or during the recruitment; (ii) routine consumption of prebiotics or probiotics in the last 3 months; (iii) history of cancer or suspected inflammatory bowel disease; (iv) diarrheal illness.

Venous blood samples were drawn using a vacutainer, and measurements of biochemical parameters were carried out. Body Mass Index (BMI) was calculated as the ratio between weight and squared height (kg/m^2^). We adopted the classifications in use by the World Health Organization (WHO): underweight-BMI under 18.5 kg/m^2^, normal weight-BMI greater than or equal to 18.5 to 24.9 kg/m^2^, overweight-BMI greater than or equal to 25 to 29.9 kg/m^2^, obesity-BMI greater than or equal to 30 kg/m^2^.

A self-questionnaire was also used to estimate the habitual dietary intake. The residents were provided with the same daily food to eat.

All the residents were further stratified according to the presence or absence of dementia. Dementia diagnoses were established clinically by the nursing home geriatrician using Italian SIGG (Società Italiana di Geriatria e Gerontologia) guidelines (equivalent to DSM-5 criteria). Individuals were classified as having dementia if they had received a clinical diagnosis of one of the following conditions: Alzheimer’s disease, Parkinson’s disease, or non-Alzheimer’s and non-Parkinson’s forms of dementia.

Frailty phenotypes were determined using hierarchical cluster analysis following Montesanto et al. (2010) [41]. We used four standardized variables: normalized Mini-Mental State Examination (MMSE) scores, residualized maximum handgrip strength (HGS), self-rated health status (SRHS), and activities of daily living (ADL) scores. Before clustering, HGS values were adjusted for age, sex, and height using ordinary least squares regression. Ward’s linkage clustering was performed on Euclidean distances. Given the different frailty profiles across age groups, participants were stratified: those aged 65–89 years were classified into three clusters (non-frail, pre-frail, and frail), while those ≥90 years were classified into two clusters (less compromised and very frail). Cluster assignment was based on a deficit score (SRHS + ADL **−** HGS − MMSE), where higher values indicated greater frailty.

### 2.2. Fecal Sample Collection and DNA Isolation

Fecal samples were collected in sterile plastic cups. Microbial DNA was extracted from feces using the Pure Link Microbiome DNA purification kit (ThermoFisher Scientifics, Monza, Italy) according to the manufacturer’s recommendations. Briefly, 0.2 g of feces was resuspended by vortexing in 600 µL of S1-Lysis Buffer and, subsequently, in 100 µL of S2-Lysis Enhancer. Samples were incubated at 65 °C for 10 min, homogenized by bead beating on a vortex for 10 min, and centrifuged at 14,000× *g* for 5 min. Then, 400 µL of the supernatants were transferred to a new microcentrifuge tube containing 250 µL of S3-Cleanup Buffer and centrifuged at 14,000× *g* for 2 min, and 500 µL of the supernatants were vortexed in 900 µL of S4-Binding Buffer. Afterwards, 700 µL of the samples were loaded onto a spin column-tube and centrifuged at 14,000× *g* for 1 min. Then, 500 µL of S5-Wash Buffer was added to each sample, and columns were centrifuged at 14,000× *g* for 1 min. Microbial DNA samples were eluted by centrifugation at 14,000× *g* for 1 min in 100 µL of S6-Elution Buffer.

### 2.3. 16S rRNA Sequencing

The variable V3–V4 region of the bacterial 16S rRNA gene was sequenced by the company BMR Genomics of Padua through the MiSeq platform (Illumina, San Diego, CA, USA). The sequence of primers used in the amplification of the samples is as follows: 341F: 5′-TCGTCGGCAGCGTCAGATGTGTATAAGAGACAGCCTACGGGNBGCASCAG-3′; 805R: 5′-GTCTCGTGGGCTCGGAGATGTGTATAAGAGACAGGACTACNVGGGTATCTAATCC-3′ [42].

### 2.4. Bioinformatic and Statistical Analysis

Raw paired-end FASTQ reads were processed in QIIME 2 (v2023.9). Amplicon Sequence Variants (ASVs) were inferred using DADA2 (v1.38.0.), including quality filtering, denoising, and chimera removal; taxonomy was assigned using the SILVA 138 reference database.

For comparisons between younger and older adults, statistical models were adjusted for age and sex. For comparisons between residents with and without dementia, models were adjusted for age, sex, nursing home residence duration, frailty status, and drugs (bisoprolol, calcium folinate, quetiapine, cholecalciferol, ferrous glycine sulfate, lactulose, pantoprazole, allopurinol, acetylsalicylic acid, metformin, ramipril, furosemide, and folic acid).

Alpha diversity (Shannon index) was computed after rarefaction to 11,300 reads per sample and compared using ANCOVA with the aforementioned covariates. Beta diversity (Bray–Curtis dissimilarity) was assessed using PERMANOVA (999 permutations), with R^2^ values reported to indicate effect size. Principal Coordinates Analysis (PCoA) was used for visualization.

Differential abundance analysis was performed using edgeR’s quasi-likelihood negative binomial model with TMM (Trimmed Mean of M-values) normalization and Benjamini–Hochberg FDR (False Discovery Rate) correction. Results were visualized as volcano plots constructed from edgeR output (log_2_ fold change and FDR-adjusted *p*-values).

Statistical significance was set at *p* < 0.05 for diversity analyses and FDR < 0.05 for differential abundance. All analyses were performed in R v4.5.1 (phyloseq, edgeR, vegan, ggplot2) and Python 3.13.2 (scipy, scikit-learn, statsmodels, pandas, numpy).

Functional profiles were predicted from 16S rRNA data in R using the Tax4Fun package.

## 3. Results

### 3.1. Participant Characteristics

In the study, we collected samples from 56 older adults (45 females and 11 males) residing in a nursing home. Anthropometric measures and biochemical parameters are shown in Table 1. All subjects have normal weight and BMI values, and although the mean biochemical parameters were within normal ranges, the relatively high standard deviations indicate substantial variability among individuals.

In Table 2, the dietary intake of the residents in the nursing home is shown. The food consumption frequency questionnaire revealed that all participants had similar eating habits consistent with a balanced Mediterranean diet, and food consumption frequencies were quite similar across all subjects.

The subjects reported being in good health, although the presence of chronic age-related disorders such as heart disease (45.5%), hypertension (34.5%), and diabetes (19.6%) was detected, all well-managed by the long-term administration of medications.

Twenty-nine older adults with dementia residing in the home were included in the study. In Table 3, the frailty status of the older adults residing in the nursing home and how it varied between demented and non-demented older adults is reported. The overall distribution of frailty categories was similar between demented and non-demented older adults (χ^2^ = 3.33, *p* = 0.344), with approximately half of participants in both groups classified as frail (51.7% in demented vs. 51.9% in non-demented). A high proportion of residents with dementia were classified as very frail (17.2% vs. 3.7%), while the non-frail category was more common among residents without dementia (29.6% vs. 17.2%).

### 3.2. Microbial Diversity in Older and Younger Adults

Across the full dataset, the total number of ASVs (features) was 5334 when comparing older adults with younger adults, with the older group averaging 247 ± 67 ASVs per sample (116–395) and the younger group averaging 156 ± 61 ASVs per sample (89–360).

**Figure 1:**
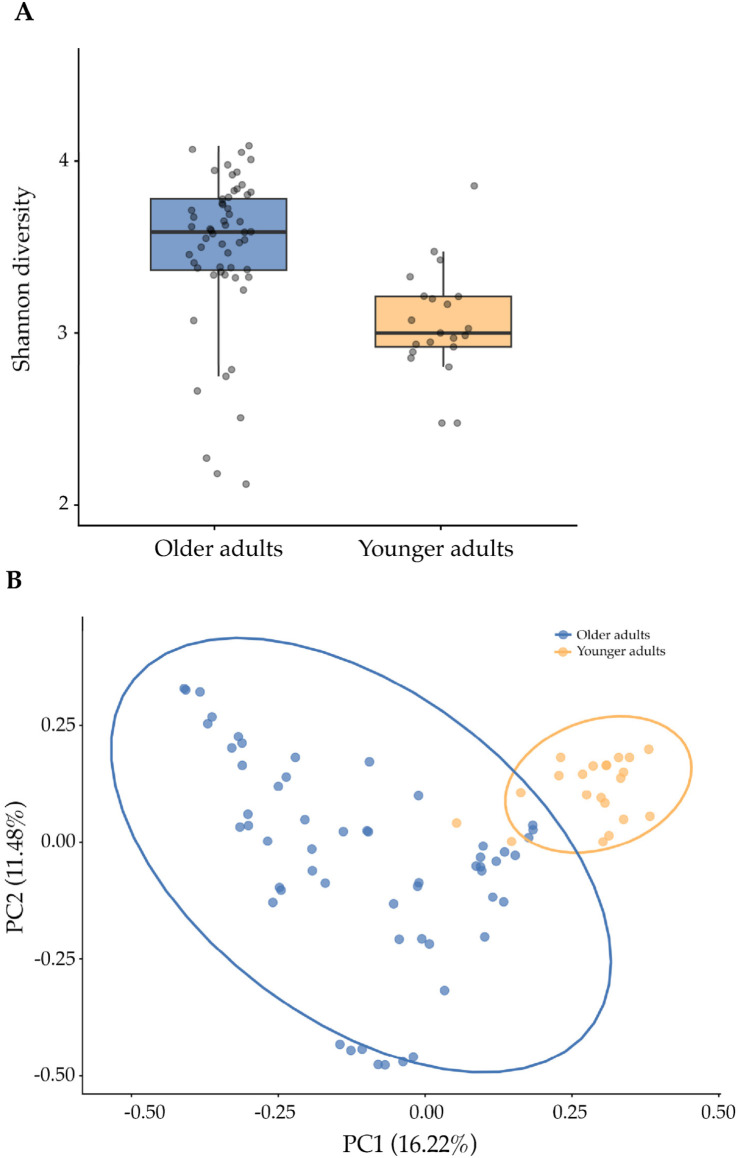
Alpha diversity evaluated by the Shannon index in older and younger adults adjusted for age and sex (**A**). Principal Coordinate Analysis of Bray–Curtis distance for beta-diversity evaluation between older and younger adults adjusted for age and sex. PC1 and PC2 represent the top two principal coordinates that captured most of the diversity (**B**).

Alpha-diversity, which evaluates intra-sample variability, was estimated using the Shannon index. The index is significantly higher in older adults compared to the younger group (*p*-value < 0.001) (Figure 1A). To assess the beta diversity between older adults and younger adults, Bray–Curtis dissimilarity was analyzed, which shows that the two groups are significantly distinct (R^2^ = 11.66%, *p*-value = 0.001) (Figure 1B).

### 3.3. Taxonomic Profiling of the Gut Microbiota

**Figure 2:**
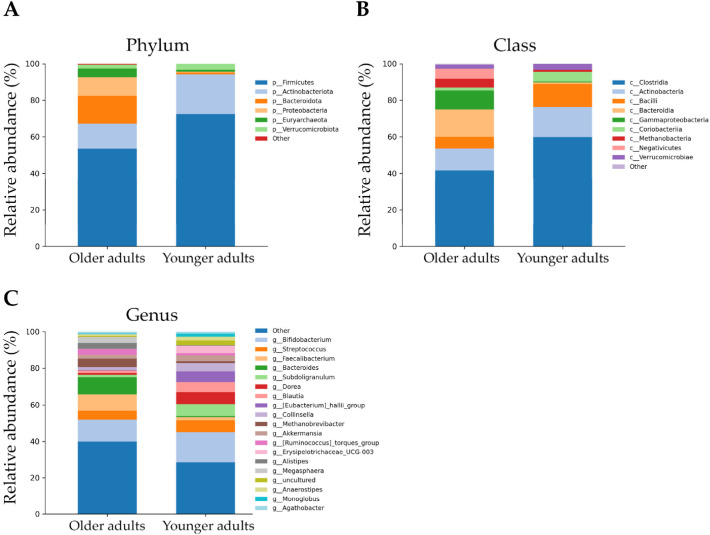
Stacked bar plots showing the composition of the gut microbiota at the phylum (**A**), class (**B**), and genus (**C**) levels in older adults compared with younger adults. Only phyla with relative abundance ≥ 1% are reported. Less abundant groups are collectively represented as Others.

At the phylum level, in older adults, *Firmicutes* dominated with a relative abundance of 53.51%, while a higher abundance was observed in younger adults at 72.41%. *Bacteroidota* and *Protoebacteria* accounted for a high proportion in the older group, at 15.04% and 10.30%, respectively, significantly higher than in the younger group (0.94% and 0.52%, respectively). The phylum *Actinobacteriota* is present in both groups, although with significantly lower relative abundance in the older (13.79% vs. 21.80%). Notably, *Euryarchaeota* were present in older adults with a relative abundance of 4.79%, whereas their abundance in the younger individuals was around 1% (Figure 2A and Table S1).

At the class level, in both groups the predominant bacteria were *Clostridia* (41.66% and 59.79%, respectively). *Bacteroidia*, *Gammaproteobacteria*, *Negativicutes*, and *Methanobacteria* were dominant in older individuals with a relative abundance of 15.04%, 10.30%, 5.53%, and 4.79%, respectively, significantly higher than in younger individuals (0.94%, 0.52%, 0.04%, and 1.04%, respectively). Relative abundance of *Actinobacteria* (11.99%), *Bacilli* (6.26%), *Verrucomicrobia* (2.14%), and *Coriobacteriia* (1.80%) were higher in the younger group (Figure 2B and Table S1) than in the older group (Figure 2B and Table S1).

At the genus level, seven genera were enriched in the older group: three from *Bacteroidota* (*Bacteroides*, *Alistipes*, *Parabacteroides*), four from *Firmicutes* (*Faecalibacterium*, *Megasphaera*, *Ruminococcus torques*, *Christensenellaceae* R7 group), and one each from *Methanobacteriota* (*Methanobrevibacter*). On the contrary, fifteen genera were less abundant in the older group: thirteen from *Firmicutes* (*Subdoligranulum*, *Streptococcus*, *Eubacterium coprostanoligenes*, *Phascolarctobacterium*, *Anaerostipes*, *Coprococcus*, *Eubacterium hallii*, *Monoglobus*, *Fusicaten-ibacter*, *Enterococcus*, *Erysipelotrichaceae_UCG-003*, *Balutia*, and *Dorea*), one from *Actinobacteria* (*Bifidobacterium*), and one from *Actynomicetota* (*Collinsella*) (Figure 2C and Table S1).

**Figure 3:**
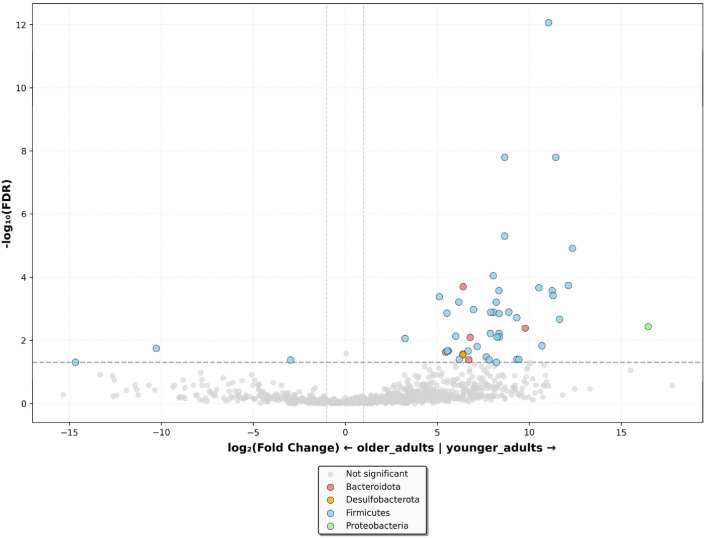
Volcano plot, showing differential microbiota at the phylum level between older adults residing in the nursing home and younger adults dwelling in the community, adjusted for age and sex. Log-transformed fold change is plotted on the x-axis, and log-transformed False Discovery Rate-adjusted *p* values (FDR) are plotted on the y-axis. FDR ≤ 0.05 and log_2_ Fold Change > 1.

By differential abundance analyses, we identified fifty species associated with age (Table S2). The volcano plot revealed that aging was negatively associated with three genera belonging to the *Firmicutes* phylum and positively associated with thirty-nine genera belonging to *Firmicutes*, five genera belonging to *Bacteroidota*, two genera belonging to *Desulfobacteriota*, and one genus belonging to *Proteobacteria* phyla (Figure 3).

### 3.4. Functional Prediction Analysis of Gut Microbiota

**Figure 4:**
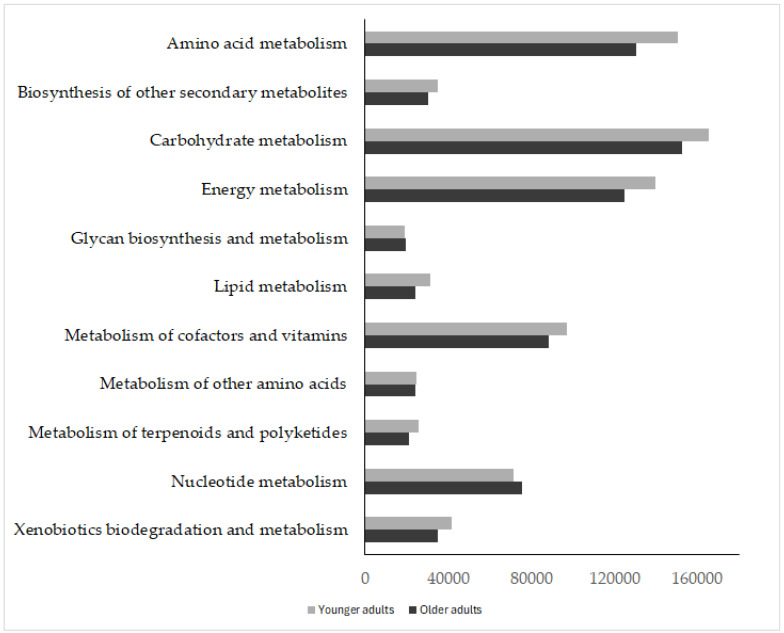
KEGG functional categories predicted from 16S rRNA profiles using Tax4Fun in older adults residing in the nursing home and in younger adults.

The Tax4Fun analytical approach was used to predict gut microbiota function in older versus younger people. KEGG pathway analysis indicated that functional categories, such as energy metabolism, carbohydrate, lipid, cofactor and vitamin metabolism, amino acids, secondary amino acids, terpenoids, and polyketides, as well as xenobiotic biodegradation and biosynthesis of other metabolites, were significantly higher in younger adults than in older adults. Conversely, the pathway involved in nucleotide metabolism was higher in the older group (Figure 4 and Table S3).

### 3.5. Alpha and Beta Diversity in Demented and Non-Demented Older Adults

**Figure 5:**
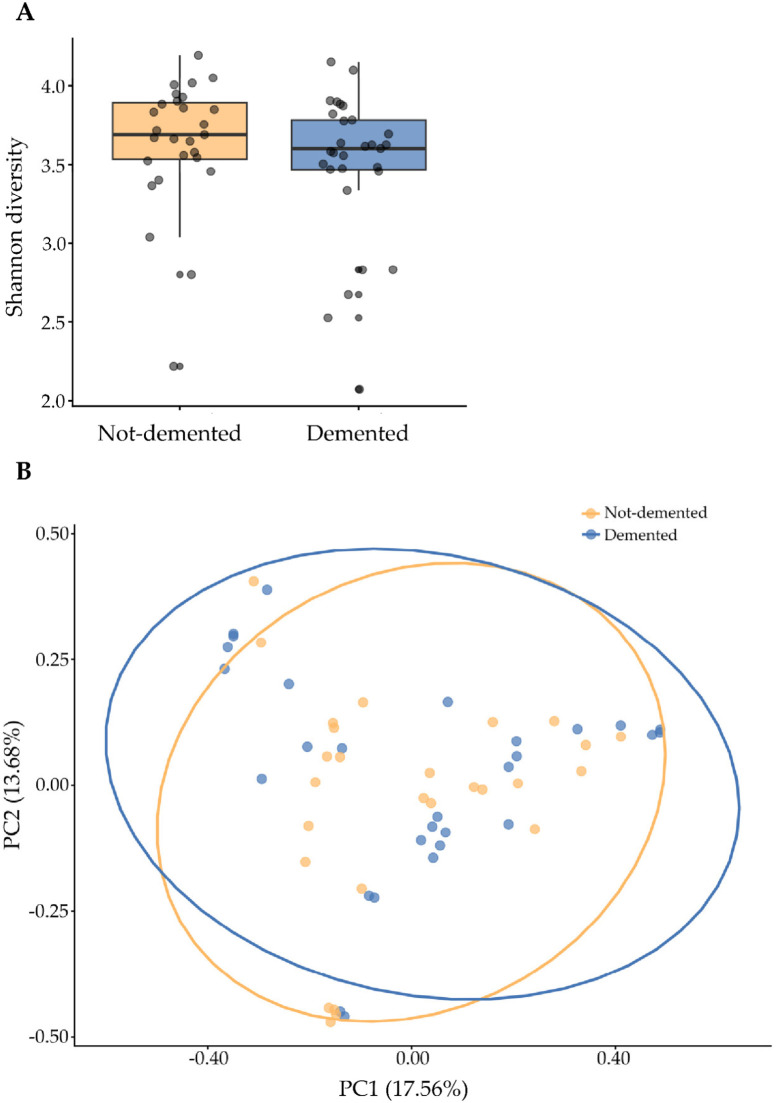
Alpha diversity evaluated by the Shannon index in demented and not-demented individuals adjusted for age, sex, frailty status, use of drugs, and time spent in the nursing home (**A**). Principal Coordinate Analysis of Bray–Curtis distance for beta-diversity evaluation, adjusted for age, sex, frailty status, use of drugs, and time spent in the nursing home, between demented and non-demented individuals (PC1 and PC2 represent the top two principal coordinates that captured most of the diversity (**B**).

A total of 4133 ASVs (features) were observed, with demented older residents of the nursing home averaging 176 ± 50 (84–265) and non-demented residents averaging 191 ± 51 (95–287). Species diversity between demented and non-demented subjects, estimated using the Shannon index, was lower in patients with dementia, although it was not statistically significant (*p*-value = 0.238) (Figure 5A). Bray–Curtis dissimilarity analysis revealed that the microbial community structures of the two groups were not significantly different (R^2^ = 1.70%, *p*-value = 0.433) (Figure 5B).

### 3.6. Taxonomic Profiling of the Gut Microbiota in Demented and Non-Demented Older Adults

**Figure 6:**
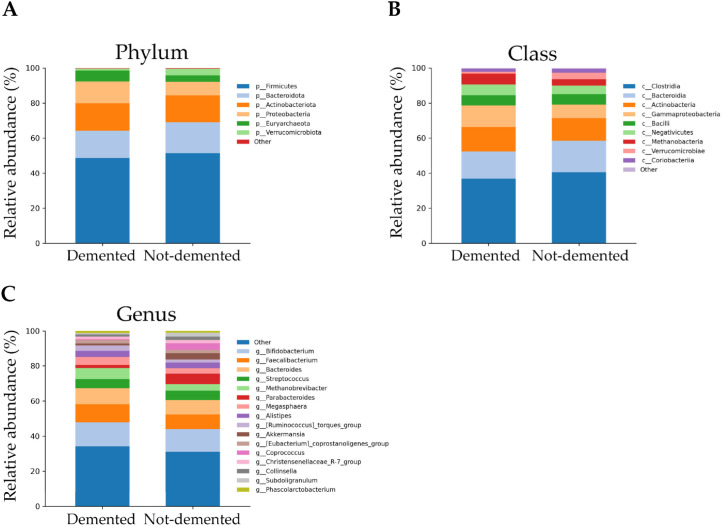
Stacked bar plots showing the composition of the gut microbiota at the phylum (**A**), class (**B**), and genus (**C**) levels in demented and non-demented older adults residing in the nursing home. Only phyla with relative abundance ≥ 1% are reported. Less abundant groups are collectively represented as Others.

Five major phyla were observed in the gut microbiota of demented older adults residing in the nursing home, classified as *Firmicutes* (48.73%), *Actinobacteriota* (15.62%), *Bacteroidota* (15.51%), *Proteobacteria* (12.39%), and *Euryarchaeota* (6.38%). More specifically, the relative abundance of *Firmicutes* and *Bacteroidota* was lower in demented than in non-demented older adults (51.38% and 17.79%, respectively). Conversely, the relative abundance of *Proteobacteria* and *Euryarchaeota* was higher in demented than in non-demented older adults (7.66% and 3.78%, respectively). *Verrucomicrobia* is present at a very low relative abundance (1.02%) in demented older adults (Figure 6A and Table S4).

At the class level, in demented individuals, the predominant bacteria were *Clostridia* (36.9%), *Bacteroidia* (15.51%), *Actinobacteria* (13.84%), *Gammaproteobacteria* (12.39%), *Methanobacteria* (6.38%), *Negativicutes* (6.03%), and *Bacilli* (5.81%). Relative abundance of *Clostridia*, *Bacteroidia*, and *Bacilli* was lower in demented than in non-demented individuals (40.66%, 17.79%, 5.98%, respectively), while *Actinobacteria*, *Gammaproteobacteria*, *Methanobacteria*, and *Negativicutes* were higher in demented than in non-demented individuals (13.03%, 7.66%, 3.78%, 4.74%, respectively). Very low abundances are observable for *Coriobacteriia* and *Verrucomicrobiae* in both demented (1.78% and 1.02%, respectively) and non-demented (2.16% and 3.74%, respectively) (Figure 6B and Table S4).

Four major genera were observed, classified as *Bifidobacterium* (13.84%), *Faecalibacterium* (10.24%), *Bacteroides* (9.21%), and *Methanobrevibacter* (6.38%), all significantly higher in demented than in non-demented older adults (13.03%, 8.25%, 8.37%, 3.78%, respectively). The least abundant taxa were *Streptococcus*, *Megasphaera*, *Alistipes*, the *Ruminococcus torques* group, the *Eubacterium coprostanoligenes* group, *Parabacteroide*, the *Christensenellaceae* R7 group, *Collinsella*, *Phascolarctobacterium*, *Dorea*, *Akkermansia*, *Subdoligranulum*, and *Coprococcus* (relative abundances between 5.1% and 0.38%) (Figure 6C and Table S4).

**Figure 7:**
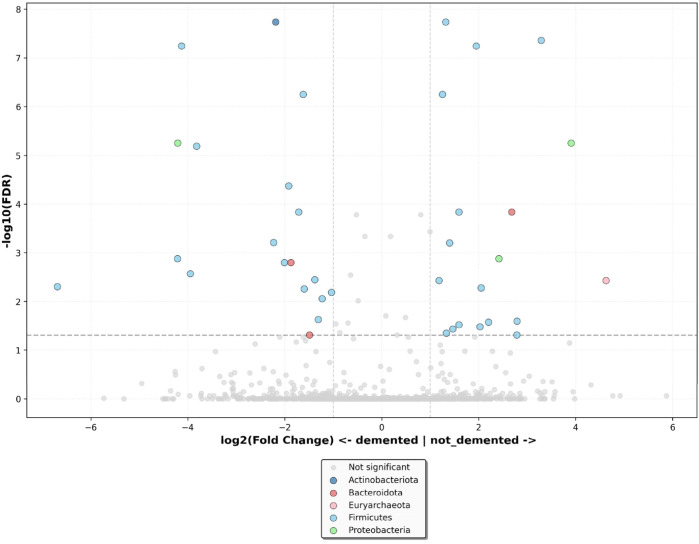
Volcano plot, showing differential microbiota at the phylum level between demented and non-demented older adults residing in the nursing home, adjusted for age, sex, frailty status, use of drugs, and time spent in the nursing home. Log-transformed fold change is plotted on the x-axis, and log-transformed False Discovery Rate-adjusted *p* values (FDR) are plotted on the y-axis. FDR ≤ 0.05 and log_2_ Fold Change > 1.

By differential abundance analyses, we identified thirty-eight species associated with dementia (Table S5). The volcano plot revealed that dementia was negatively associated with fifteen genera belonging to *Firmicutes*, two genera belonging to *Bacteroidota*, one genus belonging to *Actinobacteriota*, and one genus belonging to the phylum *Proteobacteria*. It was positively associated with fifteen genera belonging to *Firmicutes*, two genera belonging to *Proteobacteria*, one genus belonging to *Bacteroidota*, and one genus belonging to the phylum *Euryarchaeota* (Figure 7).

The STORMS checklist providing guidance for complete reporting of the study is reported in Table S6.

## 4. Discussion

Gut microbiota plays a crucial role in maintaining health. Under conditions of eubiosis, it supports longevity by modulating metabolic and immune functions, whereas dysbiosis has been associated with an increased risk of age-related diseases, including inflammatory bowel disease, musculoskeletal disorders, and metabolic and neurological disorders [42,43]. Older adults living in nursing homes represent a particularly vulnerable population due to frailty, multimorbidity, chronic illnesses, cognitive impairment, and heightened susceptibility to infections. Their relatively homogeneous lifestyle, diet, and reduced social contacts make them a valuable group for investigating age-related microbiota changes under controlled environmental conditions [44].

In this study, we analyzed the gut microbiota of 56 older adults residing in a nursing home in Calabria (Southern Italy). After adjustment for age and sex, older adults exhibited significantly higher gut microbial alpha diversity compared with younger adults, suggesting that increased microbial diversity may be associated with greater resilience and survival in later life [45]. Elevated alpha diversity is widely regarded as a marker of a healthy gut microbiota, particularly among older populations living in health-promoting environments and adhering to a healthy diet such as a Mediterranean diet [5,46]. In this regard, the structured and diversified dietary regimen provided to nursing home residents in the present study may contribute to a broader range of nutritional substrates, thereby supporting greater microbial diversity. Conversely, community-dwelling adults are more likely to follow less varied dietary patterns, often characterized by repetitive food choices, higher consumption of processed foods, sugar-sweetened beverages, and fast food, together with a lower intake of fruits and vegetables. This dietary profile is consistent with epidemiological data indicating that the Calabria region exhibits one of the highest prevalences of obesity in both pediatric and adult populations among Italian Regions (Data from Istituto Superiore di Sanità, Italian Institute for the Monitoring of public health. https://www.google.com/url?sa=t&source=web&rct=j&opi=89978449&url=https://www.epicentro.iss.it/okkioallasalute/report-regionale-2019/calabria-2019.pdf&ved=2ahUKEwi2z8nYwaSSAxV3_7sIHb6UDXcQFnoECBMQAQ&usg=AOvVaw3KOFmONsIqF2MchaIzmrjX, accessed on 22 January 2026). Furthermore, previous evidence suggests that higher alpha diversity is associated with regular physical activity in older adults, a factor that may also have contributed to the elevated microbial diversity observed in nursing home residents, who have daily access to structured exercise programs [47].

In parallel, beta diversity distances were found to be significantly different between older and younger adults, thus confirming that aging is accompanied by marked compositional changes in gut microbiota communities and supporting the notion that microbiota patterns become increasingly individualized or age-specific over time [48,49]. What emerges from our study is that both aging and institutional living contribute to shaping the gut microbiota. Older adults exhibit a recurrent age-associated microbial composition alongside taxa characteristic of nursing home residents. More specifically, a higher relative abundance of *Bacteroidota* and *Proteobacteria* phyla was observed, together with genera such as *Bacteroides*, *Faecalibacterium*, *Ruminococcaceae*, *Parabacteroides*, and *Christensenellaceae* [50]. The role of these taxa in aging is complex and, at times, controversial, as their impact may vary depending on diet, health status, and medication use. Taking together, these factors may also collectively contribute to explaining the near absence of *Bacteroidota* in younger adults dwelling in the community we observed in our study. Members of *Bacteroidota* contribute to the production of SCFAs, such as succinate, propionate, acetate, butyrate, and many proteases, supporting protein metabolism, colonization resistance by other organisms, and intestinal integrity [51]. Conversely, increased *Proteobacteria*, together with elevated levels of the species *Ruminococcus torques*, have been linked to mucus layer disruption, making it more susceptible to bacterial penetration, and, ultimately, age-associated intestinal inflammation [52]. Although *Firmicutes* and *Actinobacteriota* remained abundant in older adults, their relative abundance was reduced compared with that of younger individuals. In particular, the decline in beneficial *Firmicutes*, including members of the *Lachnospiraceae* family, aligns with observations in other aging populations, and it is often accompanied by increased *Bacteroidota* and *Proteobacteria*, a pattern associated with gut dysbiosis, frailty, and cognitive impairment [53]. The reduced *Firmicutes*/*Bacteroidota* ratio observed in our study may reflect dietary habits, pharmacological treatments, and decreased physical activity, and may plausibly contribute to sarcopenia, functional decline, and cognitive deterioration in older adults [5]. A notable finding was the decrease in *Bifidobacterium* in older subjects compared to younger ones, confirming its known decline over the lifespan [54,55]. Nevertheless, *Bifidobacterium* remained a predominant genus in older subjects, suggesting a potential compensatory or protective role, possibly mediated through anti-inflammatory activity and SCFA production. Among less commonly reported taxa, we observed a high relative abundance of *Euryarchaeota*, particularly methanogens such as *Methanobrevibacter smithii* [56,57,58]. These archaea may promote healthy aging by enhancing SCFA availability, reducing opportunistic pathogens, and lowering trimethylamine *N*-oxide (TMAO) levels [59]. The concurrent enrichment of *M. smithii* and *Alistipes onderdonkii*, together with the depletion of *Blautia luti*, may represent a compensatory mechanism counteracting the age-related decline in butyrate-producing bacteria. The exclusive presence of *Megasphaera massiliensis* in older adults is particularly intriguing. This recently identified species produces butyrate and valerate, has HDAC-inhibiting activity, and has been associated with anti-inflammatory and neuroprotective effects, highlighting its potential therapeutic relevance [60]. Similarly, the detection of *Phascolarctobacterium* exclusively in older individuals, a genus linked to propionate production and physical health, suggests a role in maintaining metabolic balance, although its effects are likely context-dependent and influenced by the overall microbial ecosystem [61].

Regarding diet, which is the most influential factor modulating the diversity and function of the gut microbiota, older adults showed good adherence to the Mediterranean diet, as reflected by the abundance of fiber-degrading and SCFA-producing genera such as *Bifidobacterium*, *Bacteroides*, *Faecalibacterium*, and *Parabacteroides.* These findings suggest that the dietary pattern implemented in the nursing home exerts a beneficial effect on gut microbial and, consequently, on residents’ health. In line with this, the low abundance of *Erysipelatoclostridiaceae* observed in our study is typically associated with fiber-rich, plant-based diets and healthier metabolic and inflammatory profiles [62,63].

Consistent with previous reports, we observed a general reduction in predicted microbial metabolic activity in older residents, likely reflecting age-related inflammation, oxidative stress, and impaired homeostasis. The increased nucleotide metabolism pathway detected in our analysis was unexpected, as this function generally declines with age, and warrants further investigation. It is plausible that a diverse, plant-rich diet may support microbial functions involved in nucleotide metabolism, thereby partially counteracting age-associated functional decline [64]. We are conscious that the functional profiles inferred using Tax4Fun are based on predictive approaches and do not represent direct measurements of microbial function. Accordingly, the results obtained through this analysis should be interpreted as hypothesis-generating and indicative of potential functional trends rather than as definitive evidence of functional activity, so no conclusive inferences on the functional role of the gut microbiota can be drawn from these data. Future investigations could be effective in overcoming the predictive nature of the functional profiles described above by determining the circulating metabolites in older adults and correlating them with the reported pathways.

Mounting evidence indicates that gut microbiota constitutes a key environmental risk factor for dementia. In the present study, participants were initially stratified according to dementia subtype; however, the resulting subgroup sizes were insufficient to support meaningful statistical analyses (Alzheimer’s disease, n = 3; Parkinson’s disease, n = 7; other forms of dementia, n = 18). Consequently, results were reported and discussed considering the dementia cohort as a whole. In a future study, to better delineate the association between gut microbiota and dementia, we plan to increase the number of study participants according to dementia type. Our findings further support the link between gut microbial alterations and cognitive impairment, in line with the concept of the gut–brain–microbiota axis. Dysbiosis driven by dietary changes, antibiotic exposure, non-steroidal anti-inflammatory drugs, and pathogenic microorganisms may influence brain function through immune, metabolic, and neuroendocrine pathways [65]. Although demented individuals showed a trend toward lower microbial diversity, our results suggest that dementia-related alterations are characterized less by a global loss of diversity and more by specific compositional shifts, with depletion of beneficial taxa and enrichment of potentially harmful ones, resulting in a distinct microbial signature. In essence, it is not only the quantity, but the composition and presence/absence of specific microbial protagonists (ASVs/taxa) that are important in distinguishing health from demented states. Indeed, microbial community structure differed between demented and cognitively healthy individuals. Dementia was associated with reduced abundance of SCFA-producing taxa, such as *Christensenellaceae*, *Lactococcus*, *Eubacterium*, and *Bacteroides*, potentially leading to diminished butyrate production and compromised gut–brain communication [66]. Conversely, we observed enrichment of several anaerobic bacterial families, including *Atopobiaceae*, *Marinifilaceae*, *Eggerthellaceae*, and *Lachnospiraceae*, which are integral components of the human gut microbiota and have context-dependent roles in health and disease [67,68]. Demented individuals also exhibited increased *Proteobacteria*, particularly *Enterobacterales*, a group repeatedly associated with neurodegeneration, amyloid burden, hippocampal atrophy, systemic inflammation, and cognitive decline [69]. Elevated *Enterobacterales*, together with reduced SCFA-producing bacteria such as *Lachnospiraceae*, may contribute to impaired intestinal barrier function and weakened neuroprotective signaling, although further investigations are still needed. The higher abundance of *Bifidobacterium* observed in our study in demented older adults is controversial in the literature, although some studies reported high levels of this microorganism associated with multiple environmental, geographical, and host-related factors [70,71,72,73]. Its high levels in demented older raises the possibility of compensatory mechanisms involving gamma-aminobutyric acid (GABA) production and microbe–host neuroactive interactions [74]. More broadly, enrichment of pro-inflammatory gut bacteria is frequently associated with elevated circulating cytokines, supporting the hypothesis that microbiota-driven peripheral inflammation may contribute to neuroinflammation and neurodegeneration.

Finally, the detection of *Pyramidobacter piscolens* in individuals with dementia is noteworthy, given its proposed role in oral-gut–brain microbial interactions [75]. Alterations in microbial composition and function may thus promote immune dysregulation and neuroinflammatory processes [76]. Taken together with evidence linking adherence to the Mediterranean diet to reduced risks of depression, anxiety, and cognitive decline, the good dietary adherence observed in this cohort may represent a protective factor, underscoring the importance of diet–microbiota interactions in promoting healthy aging and cognitive resilience.

This study provides additional insights into the composition of gut microbiota in older adults residing in a nursing home, including individuals with dementia.

Overall, our findings indicate that both aging and dementia are associated with distinct shifts in gut microbial composition, characterized by simultaneous increases and decreases across multiple genera spanning *Firmicutes*, *Bacteroidota*, *Proteobacteria*, and other phyla, suggesting that these conditions differentially modulate microbial abundance rather than inducing a uniform directional change.

Nevertheless, several limitations warrant consideration. First, the cross-sectional design precludes causal inference, rendering conclusions largely speculative. Second, the relatively small sample size may constrain the generalizability of the findings. Third, although adjustments were made for a series of factors, other potential confounding factors, including oral health, dentition status, and swallowing dysfunction, were not fully addressed. Finally, proposed biological mechanisms remain hypothetical and are not supported by experimental data, which limits the overall impact of the study.

## 5. Conclusions

Overall, our results demonstrate an age-associated remodeling of gut microbiota, characterized by the persistence of a core set of taxa commonly present in the human gut alongside distinct, subject-specific bacterial signatures. These compositional shifts likely reflect the combined influence of host-related factors as well as environmental constraints inherent to the living conditions of the studied population. On the other hand, the dementia-associated changes we report confirm the correlation between gut microbiota changes (especially the increase in inflammation-associated species) and neural dysfunction. These findings, considering the subjects’ shared environment and diet, suggest that the microbiota profiles observed in this study are specific to nursing home residents rather than generalizable to the older adult population, including community-dwelling older adults.

Our work provides a foundation for future longitudinal and mechanistic studies aimed at disentangling the relative contributions of age, vulnerability status, and environment to gut microbiota remodeling, with potential implications for targeted microbiome-based interventions in dementia. It could also be interesting to analyze the changes in the composition of the microbiota over time following a standard diet and dietary supplementation with probiotics. Furthermore, future investigations into the oral microbiota of individuals analyzed in this study are expected to strengthen the current observations regarding the gut–oral axis, providing empirical evidence to support its underlying mechanisms and reducing the speculative nature of current findings. Finally, an integration of the current study with the analysis of gut microbiota in demented older adults, with the systematic monitoring of daily food intake, food waste, and nutritional care practices, will be realized.

## Figures and Tables

**Table 1:** Characteristics of the participants to study. Data is expressed as mean standard deviation.

Variables	Mean	Standard Deviation
Weight	58.507	15.293
BMI (Kg/m^2^)	23.477	5.99
Albumin (g/dL)	5.36	7.876
Alkaline Phosphatase (U/L)	101.982	49.144
Blood Urea Nitrogen (mg/dL)	54.851	32.605
Calcium (mg/dL)	9.465	0.535
Chloride (mmol/L)	100.364	18.298
Creatinine (mg/dL)	0.946	0.553
Direct Bilirubin (mg/dL)	0.201	0.193
Ferritin (ng/mL)	157.754	102.731
Glucose (mg/dL)	95.058	23.203
GGT (U/L)	22.907	30.458
GOT/AST (U/L)	17.137	5.713
GPT/ALT (U/L)	15.729	10.819
HDL (mg/dL)	46.488	10.132
Iron (µg/dL)	51.271	21.587
LDL (mg/dL)	89.964	33.408
Potassium (mmol/L)	4.842	0.646
Sodium (mmol/L)	139.263	2.892
Total Bilirubin (mg/dL)	0.705	0.295
Total Cholesterol (mg/dL)	149.643	28.598
Triglycerides (mg/dL)	96.419	35.027
Uric Acid (mg/dL)	5.874	7.69

**Table 2:** Nutritional characteristics of the participants in the study.

Variables	Dietary Intake
Energy (kcal/day)	1750
Carbohydrate (%)	55
Protein (g/kg) *	1.2
Fate (%)	30
Vitamin B12 (μg)	4
Vitamin D (μg)	15
Calcium (mg)	1100
Iron (mg)	10
Water (Liter/day)	1.5–2

* 1.5 g/kg in patients suffering from sarcopenia.

**Table 3:** Frailty distribution in the older adults residing in the nursing home stratified by dementia status.

Frailty Categories	Demented	%	Non-Demented	%	Total
Non-frail (65–89 years)	5	17.2%	8	29.6%	13
Pre-frail (65–89 years)	4	13.8%	4	14.8%	8
Frail (65–89 or ≥90 less compromised)	15	51.7%	14	51.9%	29
Very frail (≥90 years)	5	17.2%	1	3.7%	6
Total	29	100%	27	100%	56

## Data Availability

The original contributions presented in this study are included in the article/Supplementary Material. Further inquiries can be directed to the corresponding author.

## Supplementary Materials

The following supporting information can be downloaded at: https://www.mdpi.com/article/10.3390/nu18030505/s1, Table S1. Relative abundance (%) of bacterial taxa at the phylum, class, order, family, genus, and species levels in gut samples from older and younger adults; Table S2. Differentially abundant ASVs between younger and older adults. ASVs with significant differential abundance (FDR < 0.05) were detected by the edgeR quasi-likelihood negative binomial model with TMM normalization, adjusted for age and sex. logFC: log_2_ fold change (positive = enriched in older adults; negative = depleted); logCPM: log_2_ counts per million; LR: likelihood ratio; FDR: false discovery rate. Taxonomy: SILVA 138. Table S3. KEGG level 2 functional categories predicted from 16S rRNA profiles (Tax4Fun) in older and younger adults. For each category, the Mann–Whitney U statistic (U) and *p* values are reported together with the Hodges-Lehmann estimate of the between-group location shift and its 95% confidence interval (CI, Lower, Upper). The sign of the Hodges-Lehmann estimate reflects the direction of the difference according to the group order used in the analysis; Table S4. Relative abundance (%) of bacterial taxa at the phylum, class, order, family, genus, and species levels in gut samples from demented and non-demented individuals residing in the nursing home; Table S5. Differentially abundant ASVs between nursing home residents with and without dementia. ASVs with significant differential abundance (FDR < 0.05) were detected by the edgeR quasi-likelihood negative binomial model with TMM normalization, adjusted for age, sex, frailty status, drug use, and spent time in the nursing home. logFC: log_2_ fold change (positive = enriched in dementia; negative = depleted); logCPM: log_2_ counts per million; LR: likelihood ratio; FDR: false discovery rate. Taxonomy: SILVA 138. Table S6. STORM checklist.
