# Supplementary material for: Composition of the Gut Microbiota in Older Adults Residing in a Nursing Home and Its Association with Dementia
Source: Nutrients. 2026 Feb 2;18(3):505. doi: 10.3390/nu18030505 (PMC12899124; doi:10.3390/nu18030505)
Supplement: Supplementary file 1 [file nutrients-18-00505-s001.zip › nutrients-4084277-supplementary/Legends of Supplementary Material.docx]

**Legends of Supplementary Materials**

**Table S1.** Relative abundance (%) of bacterial taxa at the phylum, class, order, family, genus, and species levels in gut samples from older and younger adults.

**Table S2.** Differentially abundant ASVs between younger and older adults. ASVs with significant differential abundance (FDR < 0.05) were detected by edgeR quasi-likelihood negative binomial model with TMM normalization, adjusted for age and sex. logFC: log₂ fold change (positive = enriched in older adults; negative = depleted); logCPM: log₂ counts per million; LR: likelihood ratio; FDR: false discovery rate. Taxonomy: SILVA 138.

**Table S3.** KEGG level-2 functional categories predicted from 16S rRNA profiles (Tax4Fun) in older and younger adults. For each category, the Mann-Whitney U statistic (U) and p values are reported together with the Hodges-Lehmann estimate of the between-group location shift and its 95% confidence interval (CI, Lower, Upper). The sign of the Hodges-Lehmann estimate reflects the direction of the difference according to the group order used in the analysis.

**Table S4.** Relative abundance (%) of bacterial taxa at the phylum, class, order, family, genus, and species levels in gut samples from demented and not demented individuals residing in the nursing home.

**Table S5.** Differentially abundant ASVs between nursing home residents with and without dementia. ASVs with significant differential abundance (FDR < 0.05) were detected by edgeR quasi-likelihood negative binomial model with TMM normalization, adjusted for age, sex, frailty status, drug use, and spent time in the nursing home. logFC: log₂ fold change (positive = enriched in dementia; negative = depleted); logCPM: log₂ counts per million; LR: likelihood ratio; FDR: false discovery rate. Taxonomy: SILVA 138.

**Table S6.** The STORMS checklist.
