# Supplementary material for: Composition of the Gut Microbiota in Older Adults Residing in a Nursing Home and Its Association with Dementia
Source: Nutrients. 2026 Feb 2;18(3):505. doi: 10.3390/nu18030505 (PMC12899124; doi:10.3390/nu18030505)
Supplement: Supplementary file 1 [file nutrients-18-00505-s001.zip › nutrients-4084277-supplementary/Table S1.docx]

**Table S1.** Relative abundance (%) of bacterial taxa at the phylum, class, order, family, genus, and species levels in gut samples from older and younger adults.

|  | **Microorganisms** | **Older adults** | **Younger adults** |
| --- | --- | --- | --- |
| **Phylum** | *Firmicutes* | 53.51 | 72.41 |
|  | *Bacteroidota* | 15.04 | 0.94 |
|  | *Actinobacteriota* | 13.79 | 21.80 |
|  | *Proteobacteria* | 10.30 | 0.52 |
|  | *Euryarchaeota* | 4.79 | 1.04 |
|  | *Verrucomicrobiota* | 2.14 | 3.28 |
|  | *Others* | 0.44 | 0.00 |
| **Class** | *Clostridia* | 41.66 | 59.79 |
|  | *Bacteroidia* | 15.04 | 0.94 |
|  | *Actinobacteria* | 11.99 | 16.53 |
|  | *Gammaproteobacteria* | 10.30 | 0.52 |
|  | *Bacilli* | 6.26 | 12.58 |
|  | *Negativicutes* | 5.53 | 0.04 |
|  | *Methanobacteria* | 4.79 | 1.04 |
|  | *Verrucomicrobiae* | 2.14 | 3.28 |
|  | *Coriobacteriia* | 1.80 | 5.27 |
|  | *Others* | 0.51 | 0.01 |
| **Order** | *Oscillospirales* | 22.28 | 15.82 |
|  | *Lachnospirales* | 16.11 | 38.94 |
|  | *Bacteroidales* | 15.04 | 0.94 |
|  | *Bifidobacteriales* | 11.99 | 16.53 |
|  | *Enterobacterales* | 10.30 | 0.52 |
|  | *Lactobacillales* | 5.31 | 7.93 |
|  | *Methanobacteriales* | 4.79 | 1.04 |
|  | *Veillonellales-Selenomonadales* | 3.88 | 0.04 |
|  | *Verrucomicrobiales* | 2.14 | 3.28 |
|  | *Coriobacteriales* | 1.80 | 5.27 |
|  | *Christensenellales* | 1.67 | 0.23 |
|  | *Acidaminococcales* | 1.65 | 0.00 |
|  | *Others* | 1.07 | 0.72 |
|  | *Erysipelotrichales* | 0.95 | 4.65 |
|  | *Peptostreptococcales-Tissierellales* | 0.65 | 2.37 |
|  | *Monoglobales* | 0.38 | 1.71 |
| **Family** | *Others* | 18.42 | 8.49 |
|  | *Lachnospiraceae* | 16.11 | 38.94 |
|  | *Ruminococcaceae* | 12.33 | 8.68 |
|  | *Bifidobacteriaceae* | 11.99 | 16.53 |
|  | *Bacteroidaceae* | 9.33 | 0.53 |
|  | *Streptococcaceae* | 4.83 | 6.66 |
|  | *Methanobacteriaceae* | 4.79 | 1.04 |
|  | *Veillonellaceae* | 3.88 | 0.04 |
|  | *Rikenellaceae* | 3.26 | 0.29 |
|  | *Oscillospiraceae* | 3.20 | 1.03 |
|  | *Tannerellaceae* | 2.26 | 0.09 |
|  | *Akkermansiaceae* | 2.14 | 3.28 |
|  | *Christensenellaceae* | 1.67 | 0.23 |
|  | *Acidaminococcaceae* | 1.65 | 0.00 |
|  | *Coriobacteriaceae* | 1.38 | 4.58 |
|  | *Eubacterium coprostanoligenes group* | 1.25 | 0.43 |
|  | *Erysipelatoclostridiaceae* | 0.71 | 4.17 |
|  | *Monoglobaceae* | 0.38 | 1.71 |
|  | *Peptostreptococcaceae* | 0.24 | 2.01 |
|  | *Enterococcaceae* | 0.19 | 1.27 |
| **Genus** | *Others* | 31.94 | 23.42 |
|  | *Bifidobacterium* | 11.99 | 16.53 |
|  | *Bacteroides* | 9.33 | 0.53 |
|  | *Faecalibacterium* | 9.04 | 1.74 |
|  | *Streptococcus* | 4.81 | 6.50 |
|  | *Methanobrevibacter* | 4.79 | 1.04 |
|  | *Megasphaera* | 3.36 | 0.00 |
|  | *Alistipes* | 3.26 | 0.29 |
|  | *Ruminococcus torques group* | 3.06 | 1.29 |
|  | *Akkermansia* | 2.14 | 3.28 |
|  | *Parabacteroides* | 1.79 | 0.09 |
|  | *Christensenellaceae R7 group* | 1.62 | 0.23 |
|  | *Collinsella* | 1.38 | 4.58 |
|  | *Subdoligranulum* | 1.29 | 6.65 |
|  | *Blautia* | 1.27 | 5.42 |
|  | *Eubacterium coprostanoligenes group* | 1.25 | 0.43 |
|  | *Phascolarctobacterium* | 1.11 | 0.00 |
|  | *UCG-002* | 1.11 | 0.69 |
|  | *Dorea* | 1.03 | 6.54 |
|  | *Agathobacter* | 1.02 | 1.01 |
|  | *Anaerostipes* | 0.89 | 2.09 |
|  | *Coprococcus* | 0.56 | 1.21 |
|  | *uncultured* | 0.51 | 2.57 |
|  | *Eubacterium hallii group* | 0.45 | 5.75 |
|  | *Monoglobus* | 0.38 | 1.71 |
|  | *Fusicatenibacter* | 0.31 | 1.13 |
|  | *Enterococcus* | 0.19 | 1.27 |
|  | *Erysipelotrichaceae UCG-003* | 0.12 | 4.01 |
| **Species** | *Others* | 78.17 | 78.36 |
|  | *Methanobrevibacter smithii* | 4.79 | 1.04 |
|  | *Megasphaera massiliensis* | 3.36 | 0.00 |
|  | *Ruminococcus torques* | 2.63 | 0.03 |
|  | *Akkermansia muciniphila* | 2.14 | 3.28 |
|  | *Human gut* | 1.94 | 0.01 |
|  | *Alistipes onderdonkii* | 1.73 | 0.01 |
|  | *Parabacteroides merdae* | 1.14 | 0.06 |
|  | *Alistipes putredinis* | 1.00 | 0.06 |
|  | *Bifidobacterium bifidum* | 0.80 | 1.12 |
|  | *Metagenome* | 0.71 | 4.59 |
|  | *Blautia luti* | 0.42 | 3.36 |
|  | *Blautia faecis* | 0.38 | 1.72 |
|  | *Dorea formicigenerans* | 0.33 | 1.61 |
|  | *Uncultured Clostridiales* | 0.26 | 3.23 |
|  | *Uncultured Eubacterium* | 0.21 | 1.52 |
