# Supplementary material for: Composition of the Gut Microbiota in Older Adults Residing in a Nursing Home and Its Association with Dementia
Source: Nutrients. 2026 Feb 2;18(3):505. doi: 10.3390/nu18030505 (PMC12899124; doi:10.3390/nu18030505)
Supplement: Supplementary file 1 [file nutrients-18-00505-s001.zip › nutrients-4084277-supplementary/Table S3.docx]

**Table S3.** KEGG level-2 functional categories predicted from 16S rRNA profiles (Tax4Fun) in older and younger adults. For each category, the Mann-Whitney U statistic (U) and p values are reported together with the Hodges-Lehmann estimate of the between-group location shift and its 95% confidence interval (CI, Lower, Upper). The sign of the Hodges-Lehmann estimate reflects the direction of the difference according to the group order used in the analysis.

|  |  |  | 95% CI for Hodges-Lehmann Estimate | | |
| --- | --- | --- | --- | --- | --- |
|  | U | p | Hodges-Lehmann Estimate | Lower | Upper |
| Xenobiotics biodegradation and metabolism | 151 | < .001 | -7333,1 | -9440 | -5069 |
| Nucleotide metabolism | 955 | < .001 | 4605,1 | 2911 | 6266 |
| Metabolism of terpenoids and polyketides | 151 | < .001 | -5006,3 | -6477 | -3440 |
| Metabolism of other amino acids | 422,5 | 0,059 | -554,9 | -1194 | 27 |
| Metabolism of cofactors and vitamins | 188 | < .001 | -9814,9 | -13161 | -5958 |
| Lipid metabolism | 159 | < .001 | -7931,1 | -10296 | -5445 |
| Glycan biosynthesis and metabolism | 657,5 | 0,43 | 267,3 | -369 | 1227 |
| Energy metabolism | 209 | < .001 | -14604,9 | -21922 | -8063 |
| Carbohydrate metabolism | 313 | 0,002 | -16448,7 | -23711 | -7106 |
| Biosynthesis of other secondary metabolites | 106 | < .001 | -4637,6 | -6203 | -3142 |
| Amino acid metabolism | 142 | < .001 | -20752,9 | -26710 | -14080 |
