# Supplementary material for: Composition of the Gut Microbiota in Older Adults Residing in a Nursing Home and Its Association with Dementia
Source: Nutrients. 2026 Feb 2;18(3):505. doi: 10.3390/nu18030505 (PMC12899124; doi:10.3390/nu18030505)
Supplement: Supplementary file 1 [file nutrients-18-00505-s001.zip › nutrients-4084277-supplementary/Table S4.docx]

**Table S4.** Relative abundance (%) of bacterial taxa at the phylum, class, order, family, genus, and species levels in gut samples from demented and not demented individuals residing in the nursing home.

|  |  | **Demented** | **Not demented** |
| --- | --- | --- | --- |
| **Phylum** | *Firmicutes* | 48.73 | 51.38 |
|  | *Actinobacteriota* | 15.62 | 15.19 |
|  | *Bacteroidota* | 15.51 | 17.79 |
|  | *Proteobacteria* | 12.39 | 7.66 |
|  | *Euryarchaeota* | 6.38 | 3.78 |
|  | *Verrucomicrobiota* | 1.02 | 3.74 |
|  | *Others* | 0.35 | 0.46 |
| **Class** | *Clostridia* | 36.9 | 40.66 |
|  | *Bacteroidia* | 15.51 | 17.79 |
|  | *Actinobacteria* | 13.84 | 13.03 |
|  | *Gammaproteobacteria* | 12.39 | 7.66 |
|  | *Methanobacteria* | 6.38 | 3.78 |
|  | *Negativicutes* | 6.03 | 4.74 |
|  | *Bacilli* | 5.81 | 5.98 |
|  | *Coriobacteriia* | 1.78 | 2.16 |
|  | *Verrucomicrobiae* | 1.02 | 3.74 |
|  | *Others* | 0.35 | 0.46 |
| **Order** | *Oscillospirales* | 21.46 | 22.37 |
|  | *Bacteroidales* | 15.51 | 17.79 |
|  | *Bifidobacteriales* | 13.84 | 13.03 |
|  | *Lachnospirales* | 12.5 | 15.11 |
|  | *Enterobacterales* | 12.39 | 7.66 |
|  | *Methanobacteriales* | 6.38 | 3.78 |
|  | *Lactobacillales* | 5.22 | 5.46 |
|  | *Veillonellales-Selenomonadales* | 4.4 | 3.16 |
|  | *Others* | 2.33 | 2.23 |
|  | *Coriobacteriales* | 1.78 | 2.16 |
|  | *Acidaminococcales* | 1.63 | 1.57 |
|  | *Christensenellales* | 1.55 | 1.93 |
|  | *Verrucomicrobiales* | 1.02 | 3.74 |
| **Family** | *Others* | 19.61 | 14.71 |
|  | *Bifidobacteriaceae* | 13.84 | 13.03 |
|  | *Ruminococcaceae* | 12.67 | 12.14 |
|  | *Lachnospiraceae* | 12.5 | 15.11 |
|  | *Bacteroidaceae* | 9.21 | 8.37 |
|  | *Methanobacteriaceae* | 6.38 | 3.78 |
|  | *Streptococcaceae* | 5.1 | 5.27 |
|  | *Veillonellaceae* | 4.4 | 3.16 |
|  | *Rikenellaceae* | 3.67 | 3.16 |
|  | *Oscillospiraceae* | 2.54 | 3.9 |
|  | *Tannerellaceae* | 2.45 | 6.19 |
|  | *Eubacterium coprostanoligenes group* | 2.17 | 1.98 |
|  | *Acidaminococcaceae* | 1.63 | 1.57 |
|  | *Christensenellaceae* | 1.55 | 1.93 |
|  | *Coriobacteriaceae* | 1.28 | 1.95 |
|  | *Akkermansiaceae* | 1.02 | 3.74 |
| **Genus** | *Others* | 33.02 | 30.1 |
|  | *Bifidobacterium* | 13.84 | 13.03 |
|  | *Faecalibacterium* | 10.24 | 8.25 |
|  | *Bacteroides* | 9.21 | 8.37 |
|  | *Methanobrevibacter* | 6.38 | 3.78 |
|  | *Streptococcus* | 5.1 | 5.27 |
|  | *Megasphaera* | 4.4 | 3.16 |
|  | *Alistipes* | 3.67 | 3.16 |
|  | *Ruminococcus torques group* | 3.13 | 1.66 |
|  | *Eubacterium coprostanoligenes group* | 2.17 | 1.98 |
|  | *Parabacteroides* | 1.75 | 5.9 |
|  | *Christensenellaceae R7 group* | 1.55 | 1.93 |
|  | *Collinsella* | 1.28 | 1.95 |
|  | *Phascolarctobacterium* | 1.14 | 0.96 |
|  | *Dorea* | 1.05 | 0.88 |
|  | *Akkermansia* | 1.02 | 3.74 |
|  | *Subdoligranulum* | 0.68 | 2.15 |
|  | *Coprococcus* | 0.38 | 3.72 |
| **Species** | *Others* | 76.76 | 74.22 |
|  | *Methanobrevibacter smithii* | 6.38 | 3.78 |
|  | *Megasphaera massiliensis* | 4.4 | 3.16 |
|  | *Ruminococcus torques* | 3.13 | 1.66 |
|  | *Alistipes onderdonkii* | 2.06 | 1.65 |
|  | *human gut* | 2.01 | 1.77 |
|  | *gut metagenome* | 1.23 | 2.68 |
|  | *Alistipes putredinis* | 1.18 | 0.98 |
|  | *Parabacteroides merdae* | 1.12 | 5.2 |
|  | *Akkermansia muciniphila* | 1.02 | 3.74 |
|  | *Bifidobacterium bifidum* | 0.72 | 1.16 |
